# A genome-wide association study for gut metagenome in Chinese adults illuminates complex diseases

**DOI:** 10.1038/s41421-020-00239-w

**Published:** 2021-02-09

**Authors:** Xiaomin Liu, Shanmei Tang, Huanzi Zhong, Xin Tong, Zhuye Jie, Qiuxia Ding, Dan Wang, Ruidong Guo, Liang Xiao, Xun Xu, Huanming Yang, Jian Wang, Yang Zong, Weibin Liu, Xiao Liu, Yong Zhang, Susanne Brix, Karsten Kristiansen, Yong Hou, Huijue Jia, Tao Zhang

**Affiliations:** 1 BGI-Shenzhen, Shenzhen,, Guangdong 518083 China; 2 China National Genebank, BGI-Shenzhen, Shenzhen,, Guangdong 518120 China; 3 BGI Education Center, University of Chinese Academy of Sciences, Shenzhen,, Guangdong 518083 China; 4 Department of Biology, University of Copenhagen, Universitetsparken 13, DK-2100 Copenhagen, Denmark; 5 Shenzhen Key Laboratory of Cognition and Gene Research, BGI-Shenzhen, Shenzhen,, Guangdong 518083 China; 6 Shenzhen Key Laboratory of Human Commensal Microorganisms and Health Research, BGI-Shenzhen, Shenzhen,, Guangdong 518083 China; 7 Shenzhen Engineering Laboratory of Detection and Intervention of Human Intestinal Microbiome, BGI-Shenzhen, Shenzhen,, Guangdong 518083 China; 8 BGI-Qingdao, BGI-Shenzhen, Qingdao,, Shandong 266555 China; 9 James D. Watson Institute of Genome Sciences, Hangzhou,, Zhejiang 310058 China; 10 Department of Biotechnology and Biomedicine, Technical University of Denmark, 2800 Kgs Lyngby, Denmark

**Keywords:** Genomic analysis, Genome-wide association studies

## Abstract

The gut microbiome has been established as a key environmental factor to health. Genetic influences on the gut microbiome have been reported, yet, doubts remain as to the significance of genetic associations. Here, we provide shotgun data for whole genome and whole metagenome from a Chinese cohort, identifying no <20% genetic contribution to the gut microbiota. Using common variants-, rare variants-, and copy number variations-based association analyses, we identified abundant signals associated with the gut microbiome especially in metabolic, neurological, and immunological functions. The controversial concept of enterotypes may have a genetic attribute, with the top two loci explaining 11% of the *Prevotella–Bacteroides* variances. Stratification according to gender led to the identification of differential associations in males and females. Our two-stage metagenome genome-wide association studies on a total of 1295 individuals unequivocally illustrates that neither microbiome nor GWAS studies could overlook one another in our quest for a better understanding of human health and diseases.

## Introduction

The gut microbiota is now recognized to play important roles in host health and diseases, affecting processes well beyond the gut^1,2^. However, owing to modulations by diet and medication, the gut microbiota is commonly viewed as highly dynamic, whereas disease markers are considered to be stable. Studies in mice^3^ and in human twins^4,5^ have observed substantial heritability for some bacteria. Several genome-wide association studies^6–10^, mostly using 16 S rRNA gene amplicon sequencing, have reported associations between host single-nucleotide polymorphisms (SNPs) and individual bacterial taxa, beta-diversity, or pathways. Yet, doubts remain as to the significance of genetic associations. For example, a recent study including a heterogeneous population of ~800 individuals reported that the average heritability of gut microbiota taxa is only 1.9%^10^. By contrast, Wang et al.^9^ identified 42 SNPs that together explained 10% of the variance of the β-diversity. Except for human sequences in the metagenomic data of the HMP (Human Microbiome Project), these studies utilized genotyping array data for host genetics and used 16 S rRNA gene amplicon sequencing except for one study^10^, in which low-depth shotgun data for fecal samples were included. The lack of high-depth whole-genome sequencing (WGS) data means that the studies rely on imputation for SNPs and could be missing potential associations from insertions/deletions (INDELs), copy number variations (CNVs), especially for rare variants. In addition, previous studies on the relation between host genome and the gut microbiota mainly investigated populations of European ancestry. Thus, how host genetics shapes the gut microbiota in Asian populations needs to be further investigated.

In this study, we identified genetic-microbial associations using for the first time high-depth sequencing data for both whole genomes and whole metagenomes, in a high-depth discovery cohort of 632 healthy Chinese individuals and a low-depth replication cohort of 663 individuals. With WGS data, we are uniquely positioned to comprehensively investigate common variants, rare variants, and CNVs associated with the gut microbiota. Twelve of the SNPs from previous studies could be broadly replicated, especially *Bacteroides stercoris*. Considering the reported gender differences in the gut microbiota^11,12^ and increasing interest in incorporating the gender perspective into metagenomic and genomic studies^13^, we carried out the first gender-specific metagenome genome-wide association studies (M-GWAS) to investigate the differences in gut microbiome-genome associations between genders. Together, our results reveal a considerable impact of host genetics on the composition and functional potential of the gut microbiota enabling the generation of a number of testable hypotheses for the association of between genetics and metagenomics in relation to diseases such as colorectal cancer and cardiometabolic diseases.

## Results

### Characteristics not reported in European cohorts

To investigate the impact of host genetics on the gut microbiota, we performed WGS on 632 blood samples to a mean depth of 44× (range from 32× to 52×) per individual, and metagenomic sequencing on 632 stool samples to an average of 8.57 ± 2.21 GB (Supplementary Fig. S1a, b and Table S1). This 4D-SZ discovery cohort had a mean age of 30.7 ± 5.5 years (mean ± s.d., range of 6–35 years), a mean body mass index (BMI) of (21.8 ± 6.3) and 53.5% were females (Supplementary Table S2). We observed in this Chinese cohort that each genome differs between one another by 3.9–4.9 million sites (Supplementary Table S3). Variants were directly determined from the high-depth human genomes, including 38 million SNPs, 5 million INDELs, and 40 thousand CNVs. In all, 6.5 millions of these were common variants (minor allele frequency (MAF) > 0.05), and 36.5 million were rare and low-frequency variants (MAF) ≤ 0.05). Taxonomic profiling of the fecal metagenomes resulted in 19 phyla, 21 classes, 40 order, 77 families, 307 genera, and 519 species. The top five abundant phyla in this cohort were Bacteroidetes (relative abundance of 51.0% ± 13.5%), Firmicutes (11.2% ± 5.6%), Proteobacteria (2.8% ± 3.7%), Fusobacteria (0.3% ± 1.1%), and Actinobacteria (0.13% ± 0.27%) (Supplementary Fig. S2). Based on existing knowledge, we performed all M-GWAS by including covariates for gender, age, BMI, diet and lifestyle factors, stool form, defecation frequency, as well as the top four PCs to account for the population structure (Supplementary Table S2, and Materials and methods).

**Fig. 1:**
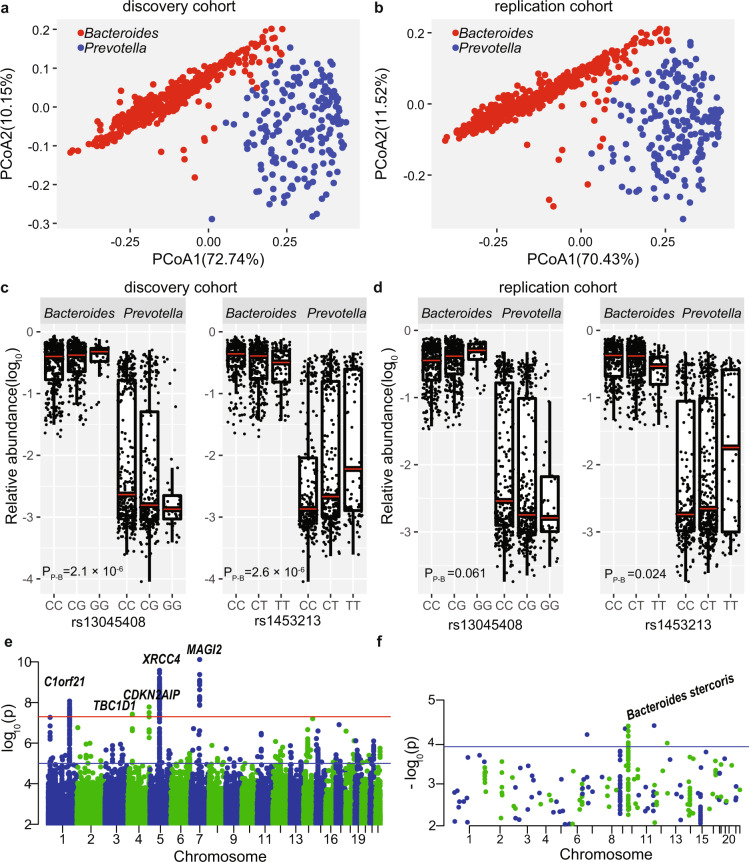
Identifying host genetic variants associated with microbiome enterotypes and principal coordinates (PCoAs, computed using Bray–Curtis dissimilarity). **a** The enterotype plot of 618 individuals in discovery cohort. Two clusters were shown with red dots representing *Bacteroides*-dominated enterotype (440 individuals) and blue dots representing *Prevotella*-dominated enterotype (178 individuals). The first two principal components (PCoA1 and PCoA2) are shown, with the amount of variation explained are reported for each axe. **b** The enterotype plot of 663 individuals in replication cohort. Two clusters were shown with red dots representing *Bacteroides*-dominated enterotype (473 individuals) and blue dots representing *Prevotella*-dominated enterotype (190 individuals). **c** The minor allele G of SNP rs13045408 at *BTBD3-LINC01722* were positively correlated with *Bacteroides* abundance and negatively correlated with *Prevotella* abundance in discovery cohort. However, SNP rs1453213 at *OXR1* had opposite effect effects to enterotypes compared with that of rs13045408. **d** rs13045408 and rs1453213 associated with “*Bacteroides*-*Prevotella*” enterotype in replication cohort (*PP-B* = 0.061 and 0.024, respectively). **e** Manhattan plots of the host genetic variants associated with microbiome β-diversity (computed as Bray–Curtis dissimilarity matrix). The red line represents a genome-wide significant *P* value (5 × 10^–8^) and blue line represents suggestive *P* value (10^−5^). Five top loci were marked with gene name. **f** The replicated *P* value in this study for the 391 SNPs previously reported to be significantly associated with the microbiome. 12 SNPs are successfully replicated at *P* 1.7 × 10^−4^ = 0.05/296 (blue line), nine of which were most associated with *Bacteroides stercoris*.

Unlike M-GWAS using chip data on European cohorts, we identified suggestive host genetic associations in relation to enterotypes following the enterotype classification approach recommended by Costea et al.^14^ (Fig. 1, Supplementary Table S4). Principal coordinate analysis (PCoA) as well as Dirichlet multinominal mixture (DMM) model using Bray–Curtis dissimilarity showed that the microbiomes of this Chinese cohort could be represented by two clusters dominated by *Bacteroides* and *Prevotella*^15^, containing 440 and 178 individuals, respectively (Fig. 1a). The existence of a *Prevotella* driven enterotype possibly reflected the higher prevalence of *Prevotella* in developing countries^16,17^. The top two loci associated with the *Bacteroides*–*Prevotella* dichotomy (*P*_*P-B*_ = 2.08 × 10^−^^6^ and *P*_*P-B*_ = 2.6 × 10^−6^, respectively, using *Prevotella* as cases and *Bacteroides* as controls in a logistic regression model) together explained 11% (standard error (SE) = 6%, *P* = 8.47 × 10^−^^10^ using likelihood ratio test) of the variance of the *Bacteroides* versus *Prevotella* enterotype. Despite a report challenging the negative association between *Bacteroides* and *Prevotella*^18^, owing to the statistically well-known loss of one degree of freedom in compositional data^19^, genetic associations for these genera also showed the opposite trend. The minor allele of the top SNP, rs13045408 at *BTBD3*-*LINC01722*, positively correlated with *Bacteroides* abundance (*β* = 0.043, *P* = 5.3 × 10^−^^3^) and negatively correlated with *Prevotella* abundance (*β* = −1.76, *P* = 1.6 × 10^−^^4^) (*P*_*P-B*_ = 2.1 × 10^−^^6^, Fig. 1c); on the other hand, the minor allele of the other SNP rs1453213 at *OXR1* positively correlated with *Prevotella* (*β* = 2.23, *P* = 1.3 × 10^−^^7^) and negatively correlated with *Bacteroides* (*β* = −0.049, *P* = 3.4 × 10^−^^4^) (*P*_*P-B*_ = 2.6 × 10^−^^6^).

In order to replicate these suggestive associations, we sequenced a replication cohort of 663 individuals (metagenomic shotgun sequencing for stool samples to an average of 8.59 ± 2.14 GB, but 7× WGS for human genomes (range from 5× to 12×, Supplementary Table S1 and Fig. S1c, d)). Summary statistics of the covariates was largely similar (Supplementary Table S2). This replication cohort comprised 473 *Bacteroides*-dominated and 190 *Prevotella*-dominated individuals (Fig. 1b). The top two associations for the *Bacteroides*-*Prevotella* dichotomy remained (Fig. 1d, *P*_*P-B*_ = 0.024 for rs1453213 in *OXR1* and *P*_*P-B*_ = 0.061 for rs13045408 at *BTBD3*-*LINC01722*).

We next investigated associations between genetic variation and microbiome β-diversity. This analysis found five loci with marginal genome-wide significance (*P* < 5 × 10^−8^, Fig. 1e, Supplementary Table S5). Three SNPs, rs60689247 in *MAGI2*, rs7716962 in *XRCC4*, and rs61823500 in *C1orf21*, are located in the intronic region of the genes. *MAGI2* is related to multiple phenotypes or diseases in the GWAS catalog^20^, including BMI, schizophrenia, coronary artery calcification and type 2 diabetes. The protein encoded by *XRCC4* functions together with DNA ligase IV and the DNA-dependent protein kinase in the repair of DNA double-strand breaks. The other two SNPs, rs11732767 and rs1967284 are located in the intergenic regions of *CDKN2AIP* and *TBC1D1*, respectively. *CDKN2AIP* is critical for the DNA damage response and *TBC1D1* has been linked to Crohn’s disease, and lymphocyte count. These are interesting associations, given the increasing incidences of Crohn’s disease and cancer. The association between rs61823500 at *C1orf21* and β-diversity could be replicated (*P* < 0.05) both in our replication cohort and a German cohort^9^. However, the three previous studies^4,9,10^ identified a total of 64 SNPs associated with beta-diversity of the gut microbiota. Of these, only one SNP was replicated here with nominal significance (*P* = 0.013, Supplementary Table S6), and none was significant after multiple-test correction. Eight of the 64 SNPs were not found or rare in the Chinese population (MAF < 0.01). The allele frequencies of these 64 SNPs differed significantly between the Chinese and the European populations (*t*-test *P*_*difference*_ = 1.55 × 10^−5^, Supplementary Fig. S3). 391 SNPs have been previously reported to associate with specific taxa, and 95 of the 391 SNPs were not found or rare in Chinese population. We were able to replicate 12 of the 296 reported associations at the phylum level (*P* < 0.05/296 = 1.7 × 10^−4^, Fig. 1f, Supplementary Table S7), especially the association with *Bacteroides stercoris*^10^. In summary, huge population heterogeneity exists, as also known from GWAS studies^21^, and it is necessary to identify Asian-specific host genome–microbiome associations for better understanding genome–microbiome interactions among different ethnicities.

### Common variants M-GWAS identifying abundant genetic signals for the gut microbiome

**Fig. 2:**
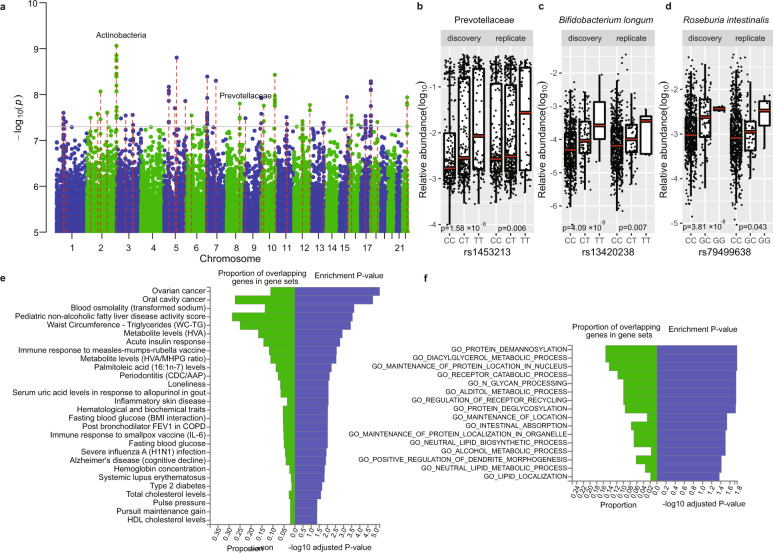
M-GWAS results for the gut microbiome and functional analysis. **a** Manhattan plot showing the associations between common variants and taxa, only suggestive associations with *P* < 10^−5^ were showed. The gray line represents a genome-wide significant *P* value (5 × 10^−8^). The red lines showed the 37 loci reaching the genome-wide significance. Two high heritable taxa, Actinobacteria linked to rs13420238 at *LOC150935* and Prevotellaceae linked to rs1453123 at *OXR1*, were marked. **b** rs1453123 at *OXR1* associated with Prevotellaceae both in discovery (*P* = 1.58 × 10^−8^) and replication (*P* = 0.006) cohort. **c** rs13420238 at *LOC150935* associated with *Bifidobacterium longum* (Actinobacteria) both in discovery (*P* = 4.09 × 10^−9^) and replication (*P* = 0.007) cohort. **d** rs79499638 near *PLXDC2* associated with *Roseburia intestinalis* both in discovery (*P* = 3.81 × 10^−8^) and replication (*P* = 0.043) cohort. **e** Enriched traits or diseases identified in GWAS catalog using 109 mapped genes from 37 significant loci. **f** Enriched KEGG or GO pathways identified for 109 mapped genes from 37 significant loci.

To detect associations between the gut microbiome and specific genetic variants, we first performed common variants M-GWAS using a linear model for microbial taxa present in over 95% of the individuals, and a logistic model for zero-inflated microbial taxa present in over 10% of the individuals (Supplementary Table S8). We identified 320 significant associations involving 37 loci and 51 bacterial taxa (*P* < 5 × 10^−8^, Fig. 2a, Supplementary Table S9). Our discovery GWAS was performed in a manner consistent with good power given our sample sizes, MAF, and effect size (Supplementary Table S10). The strongest signal (*P* = 1.68 × 10^−9^, Supplementary Fig. S4a, b) was observed for the phylum Actinobacteria and its members, including class Actinobacteria, family Bifidobacteriaceae, genus *Bifidobacterium*, species *Bifidobacterium longum* and *Bifidobacterium* *breve* (Fig. 2a). Actinobacteria associated with SNP rs62183161 in the *LOC150935* gene, which has been reported to be linked to body composition measurement and energy intake^22^. Prevotellaceae was associated with SNP rs1453123 in the *OXR1* gene (oxidation resistance 1, *P* = 1.58 × 10^−8^, Supplementary Fig. S4c, d), encoding the protein, which controls the sensitivity of neuronal cells to oxidative stress and lack of Oxr1 caused cerebellar neurodegeneration in mice^23^. Our results are consistent with the UK twins’^5^ and Korean twins’ studies^24^ which reported that the genera *Prevotella* (*h* = 0.57) and *Bifidobacterium* (*h* = 0.457) had high heritability. *Eggerthella* abundance was associated with a missense variant rs3749147 in the *GPN1* gene (*P* = 3.2 × 10^−8^). Notably, rs3749147 has been reported to associate with the levels of serum triglyceride, gamma-glutamyl transferase, albumin, and creatinine and several diseases, including urolithiasis, type 2 diabetes (T2D), esophageal cancer, schizophrenia, ischemic stroke^25^ (Supplementary Table S11). rs3749147 was also correlated with waist circumference and triglycerides in the GWAS catalog and the *GPN1* gene has been linked to oral cavity cancer, palmitoleic acid levels and periodontitis (Supplementary Table S12). In addition to the *GPN1* locus, rs142489578 in the *ARAP1* gene, associated with *Erwinia amylovora*, rs11236524 near the *MOGAT2* gene, associated with *Bacteroides plebeius*, and rs2419580 in the *RBM20* gene, associated with *Actinomyces odontolyticus*, were also linked to T2D. SNP rs2902875 in the *MIR4422HG* gene associated with *Simonsiella muelleri* was also associated with low density lipoprotein cholesterol, and rs4714598 in the *TRERF1* gene, associated with unclassified *Prevotella* sp. oral taxon 317 has also been associated with Eosinophil cell count in both the BioBank Japan and GWAS catalog studies. Taken together, 27 and seven of the 37 genome-wide significant loci have been reported to associate with traits or diseases in the BioBank Japan and GWAS catalog, respectively (Supplementary Tables S11 and S12).

Among the 320 associations involving the 37 loci identified in the discovery cohort, 220 associations involving 10 loci were covered by the low-depth replication dataset. We were able to replicate 3 of the 10 loci with the same effect (*P* < 0.05): rs13420238 in *LOC150935* had a *P* = 0.007 with *B. longum*, rs1453123 in *OXR1* had a *P* = 0.006 with Prevotellaceae and rs79499638 near *PLXDC2* had a *P* = 0.043 with *Roseburia intestinalis* (Fig. 2b–d, Supplementary Table S9).

Functional annotations using the FUMA tool^26^ further showed that the 37 loci mapped to 109 genes, which are associated with three main traits and diseases in the GWAS catalog^20^ (false discovery rate (FDR) adjusted *P* < 0.05, Fig. 2e): (1) metabolism related traits: waist circumference—triglycerides, metabolite levels (homovanillic acid), serum uric acid levels in response to allopurinol in gout, acute insulin response, fasting blood glucose, total cholesterol levels and HDL cholesterol levels; (2) immune-related diseases: inflammatory skin disease, systemic lupus erythematosus, and type 2 diabetes; (3) nervous system related disease: Alzheimer’s disease (cognitive decline) and loneliness. Furthermore, we performed gene set enrichment analyses and identified 16 significantly enriched KEGG or GO terms after FDR correction (*P* < 0.05, Fig. 2f), including multiple metabolic process such as propanoate, fatty acid, diacylglycerol, and alditol metabolism, as well as neutral lipid biosynthetic processes.

In addition, we investigated genetic variants that correlated with the functional capacity of the gut microbiota according to gut metabolic module (GMMs)^27^. We found eight loci significantly associated with seven GMMs (*P* < 5 × 10^−8^, Supplementary Table S13). The strongest association was identified for maltose degradation and nine SNPs (*P* = 4.5 × 10^−9^) in the *SLC41A2* gene encoding the solute carrier family 41 member 2, involved in transport of glucose and other sugars, bile salts and organic acids, metal ions and amine compounds. We also found genetic signals for butyrate production and mucin degradation. Mucin degradation has been implicated in metabolic regulation, obesity and type 2 diabetes. Three SNPs near the *CCR3* gene associated with mucin degradation and the *CCR3* gene has been associated with obesity-related traits and coronary artery disease (*P* < 1.0 × 10^−8^) in a transcriptome-wide association study^28^. Our results suggest that mechanistic investigations of these SNPs should take the gut microbiome into consideration.

### Rare variants- and CNVs-based M-GWAS further reveal genetic impact on the gut microbiome

Taking advantage of the high-depth whole-genome and metagenome sequencing data, we considered whether rare variants in any gene and copy number variants contributed to the gut microbiota composition. We tentatively identified 60 associations involving 47 genes and 54 bacterial taxa (*P* < 2.14 × 10^−6^ = 0.05/27874 for Bonferroni correction of 27,874 individual genes, Supplementary Table S14), including the *PCSK9* gene, a target for lowering LDL cholesterol. We evaluated the interaction between proteins encoded by the 47 genes by constructing protein–protein interaction (PPI) networks. We found that 34 of the encoded proteins participated in the network (Supplementary Fig. S5). These 34 proteins exhibited more interactions than expected for a random set of proteins of similar size (enrichment *P* = 0.037), indicating a functional intersection of the 34 microbiome-associated proteins. KEGG pathway analysis of the 34 genes showed enrichment in two main pathways (Supplementary Table S15), including hsa03030:DNA replication (FDR = 0.002) and hsa01100:Metabolic pathways (FDR = 0.044).

CNVs-based M-GWAS identified 18 CNVs associated significantly with 20 bacterial taxa (*P* < 6.25 × 10^−6^ = 0.05/8000 for Bonferroni correction of 8 K common CNVs with MAF > 0.01, Supplementary Table S16). In all, 13 of these CNVs overlap with CNVs recorded in the Database of Genomic Variants (DGV)^29^. Eight CNVs reside within genes, mainly in intronic regions. We found that the butyrate-producing bacterium SS3/4 associated with a 28.7 kb CNV region (chr4:69384168−69412841, *P* = 3.1 × 10^−6^, frequency = 0.03) located 67 kb downstream of the *UGT2B4* gene. The CNV includes many variants, which involved in expression quantitative trait loci and regulated the expression of *UGT2B4* in heart tissue and *UGT2B28* in the esophagus mucosa and liver (Supplementary Table S17), consistent with functions of butyrate or other bacterial metabolites in these tissues. Moreover, one SNP rs12505338 and three other SNPs in the CNV region have been associated with serum concentration of stearate (18:0) (*P* = 9.3 × 10^−5^) and glutamate (*P* = 7.3 × 10^−6^), respectively (Supplementary Table S18), according to the NHLBI GRASP catalog^30^. Thus, associations between the gut microbiome and rare variants and CNVs may also have important functional implications in relation to host physiology.

Common variants-, rare variants-, and CNVs- based associations separately explained 8.3%, 11.4%, and 4.9% of the microbiome composition (Supplementary Table S19). Combined they explained 20.6% of the microbiota composition. In addition, the average occurrence rate of gut microbiome taxa associated with common variants or rare variants were 0.768 and 0.596, respectively (Supplementary Tables S9 and S14, Wilcoxon test, *P* = 0.008). These results indicate that rare host variants also contribute to shaping of the gut microbiome, especially for less-common members of the community.

### A gene-bacteria axis in gender-differential metabolic and neuronal functions

In all, 53.5% of this cohort were female, permitting a comparison between sexes. Females showed higher alpha diversity than men (Wilcoxon test, *P* < 0.05, Supplementary Fig. S6), and we identified 32 taxa that differed significantly between sexes in discovery cohort, 27 of which were consistently validated in the replication cohort (FDR *q* < 0.1 using MaAslin, Supplementary Table S20). Phylum Actinobacteria and its members, including class Actinobacteria, order Bifidobacteriales, family Bifidobacteriaceae, genus *Bifidobacterium* were all significantly enriched in females. By contrast, *Fusobacterium* was significantly enriched in males.

**Fig. 3:**
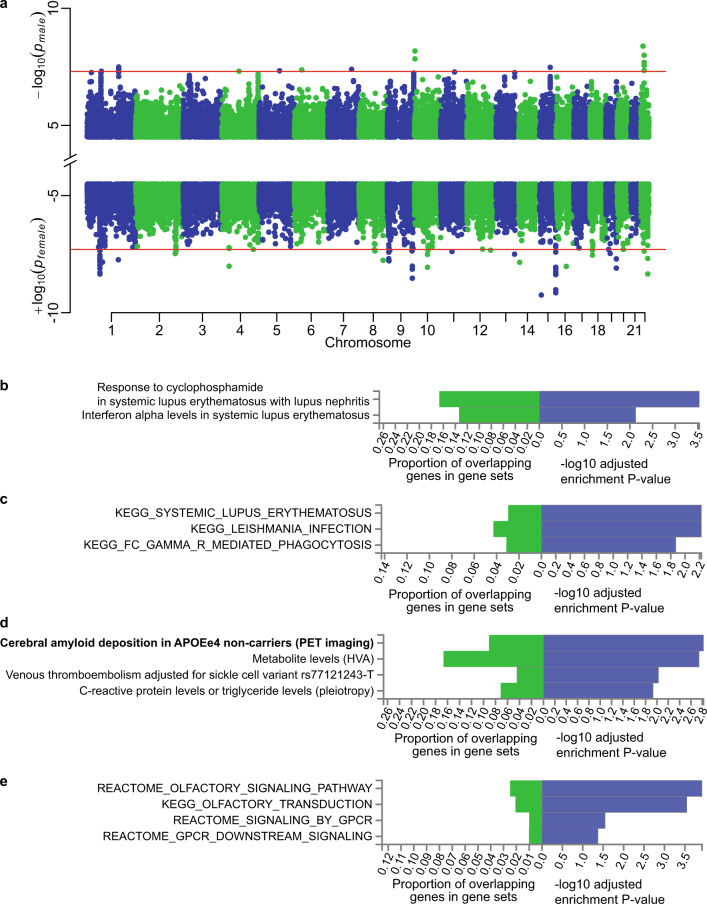
Gender-specific associations and functional analysis. **a** Manhattan plot showing the male-specific and female-specific associations for gut microbiome. The red line represents the genome-wide *P* threshold (5 × 10^−8^). **b** Enriched traits or diseases identified using male-specific associations mapped in GWAS catalog. **c** Enriched KEGG, REACTOME, or GO pathways identified for male-specific associations using FUMA tool. **d** Enriched traits or diseases identified using female-specific associations mapped in GWAS catalog. **e** Enriched KEGG, REACTOME, or GO pathways identified for female-specific associations using FUMA tool.

Since the gut microbiome exhibited striking difference between males and females, we performed a sex stratified association analysis of host genetic variants and gut bacteria, the 37 associations were overlapped between genders (Supplementary Table S21, *P* < 0.05 both in males and in females, and *P* < 5 × 10^−8^ in combined results), identical to the combined analysis (Supplementary Table S9). Especially, we identified 33 male-specific (*P* < 5 × 10^−8^ in males but *P* > 0.05 in females) and 37 female-specific associations (*P* < 5 × 10^−8^ in females but *P* > 0.05 in males) linked to gut bacteria (Fig. 3a and Supplementary Table S22). We compared the effect sizes of identified variants between genders, and confirmed that all the variants showed a significant difference (*P*_*difference*_ < 0.01). Five loci of the 70 associations linked to traits or diseases in GWAS catalog (Supplementary Table S23). Rs4650205 in *NEGR1-LINC01360* gene significantly associated with the abundance of genus *Acidaminococcus* in males (*P* = 4.87 × 10^−8^) but not in females (*P* = 0.48), and its proxy SNPs (linkage disequilibrium *r*^2^ > 0.6) were reported linked to multiple nervous system disorders such as autism spectrum disorder, schizophrenia, depression and migraine by substantial GWAS studies. Female-specific SNP rs61781314 in *LEPR* gene was associated with both genus *Eggerthella* and species *Eggerthella lenta*, and its proxy SNP rs17415296 linked to blood protein levels (*P* = 4 × 10^−2 29^). *Eggerthella lenta* as an opportunistic pathogen have been reported to underlie human infections and enriched in T2D^31^, rheumatoid arthritis (RA)^32^ and atherosclerotic cardiovascular disease (ACVD) patients^33^. The protein encoded by *LEPR* is a receptor for leptin (an adipocyte-specific hormone that regulates body weight), and is also involved in the regulation of fat metabolism and pituitary dysfunction. In the low-depth replication cohort (327 males and 336 females), associations including the male-specific association of rs6871146 with *Lactococcus* (Supplementary Table S22, *β*_*male*_ = 0.42 and *P*_*male*_ = 0.027, *β*_*female*_ = −0.47 and *P*_*female*_ = 0.062) and the female-specific association of rs7165633 and *Mobiluncus mulieris* (*β*_*male*_ = −0.009 and *P*_*male*_ = 0.96, *β*_*female*_ = 0.48 and *P*_*female*_ = 0.008) were replicated. The female-specific association with *Mobiluncus* suggests an intestinal reservoir for the bacterium which is involved in vaginal infections^34^.

We investigated the overlapped genes between gender-specific genes and traits or diseases-associated genes in GWAS catalog (Fig. 3b–e), then found that genes located in female-specific loci enriched in four phenotypes, including two metabolic traits, i.e., metabolite levels (homovanillic acid) and C-reactive protein levels or triglyceride levels (pleiotropy). Genes located in male-specific loci enriched in mainly the systemic lupus erythematosus related traits. Interestingly, one locus chr19:53772987-53796549 was both male and female-specific locus although associated with different taxa in different genders, and this locus located nearby the gene family *MIR371A-MIR372-MIR373*. WikiPathway analysis showed this gene family related to “miRNAs involved in DNA damage response”, suggesting that gut bacteria may participated in DNA damage response in both genders. In addition, gender-specific loci were also enriched in the pathway “leptin insulin overlap”, consistent with the association between *LEPR* gene and *Eggerthella lenta* as described above. Moreover, gene set enrichment analysis identified 37 female-specific loci involved in pathways “olfactory signaling” and “signaling by G-protein-coupled receptor (GPCR)”, females had been reported superior to males in olfactory abilities^35^ and had higher levels of G-protein-coupled kinases [GPCR kinase (GRK)] 3 and 5 than male^36^. Male-specific loci related to pathways “systemic lupus erythematosus” and “leishmania Infection”. Taken together, the gut microbiome exhibited differential associations with the human genome in males and females, and might contribute to different metabolic and neuronal functions as well as disease susceptibility.

### M-GWAS helps understand biomarkers from MWAS

**Fig. 4:**
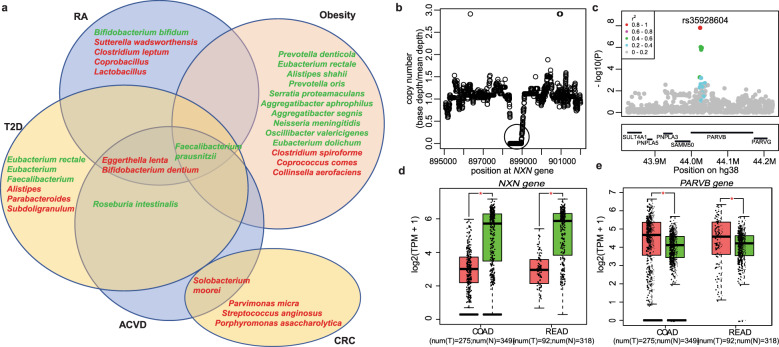
M-GWAS helps understand biomarkers from MWAS. **a** Venn diagram showing the bacteria that identified associated with genetic variants and were reported in multiple MWAS studies, including type 2 diabetes (T2D), rheumatoid arthritis (RA), atherosclerotic cardiovascular disease (ACVD), colorectal cancer (CRC), and obesity. Bacteria in overlapping areas indicates it was reported in two or more MWAS studies. The bacteria marked in red represents enrichment in cases, and green represents enrichment in controls. **b** A copy loss of 642 bp (marked by green circle) in *NXN* gene was detected in Chinese individuals. **c** Box plots of the *NXN* gene expression in COAD and READ cases compared with normal tissue samples using GEPIA tool. *COAD* colon adenocarcinoma, *READ* rectum adenocarcinoma. **d** Region plot of the most significant loci for *PARVB* gene. Each point represents a SNP or INDEL and is colored with the *r*^2^ value as calculated in this cohort. The lead SNP rs35928604 (*P* = 1.55 × 10^−8^) is highlighted with red. **e** As in **c**, box plots of the *PARVB* gene expression in COAD and READ cases compared with normal tissue samples.

We note that our M-GWAS discovered signals for some of the bacteria often reported from metagenome-wide association studies (MWAS)^1^, e.g. three butyrate-producing species, *R. intestinalis*, *Eubacterium rectale* and *Faecalibacterium prausnitzii*, have been associated with healthy controls in MWAS for T2D, ACVD and obesity as well as *Alistipes shahii* associated with lower BMI^31,33,37^ (Fig. 4a). We observed the species *R. intestinalis* associated with rs79499638 (*P* = 3.81 × 10^−8^) and rs760646544 (a insertion of CTGTT, *P* = 1.75 × 10^−8^) near *PLXDC2* (related to nidogen-1 measurement and diabetic retinopathy in GWAS catalog). Species *Eubacterium rectale* negatively associated with rs1555188 near *PHF21B* in females (*P* = 4.52 × 10^−9^) but not in males (*P* = 0.55). Genus *Faecalibacterium* and species *F. prausnitzii* were identified linked to *DYNLL1* gene (*P* = 8.83 × 10^−8^) which included 94 rare variants in gene-based association analysis. *Alistipes shahii* associated with rs72627489 near *SOWAHC* in gender-combined analysis (*P* = 8.58 × 10^−9^) and rs914338 near *UNC93A* in male-specific analysis (*P* = 2.40 × 10^−8^). We confirmed the association between *R. intestinalis* and rs79499638 and rs760646544 near *PLXDC2* in replicate cohort (*P* = 0.043, Fig. 2d, Supplementary Table S9). Consistently, the abundance of *R. intestinalis* showed higher correlation in monozygotic compared to dizygotic twins from the United Kingdom^5^.

*Bifidobacterium dentium*, enriched in RA^32^, ACVD as well as schizophrenia patients. CNVs-based M-GWAS identify the association between *Bifidobacterium dentium* and nucleoredoxin (*NXN*) with copy loss of 642 bp (chr17:898377−899018, *P* = 4.11 × 10^−6^, Fig. 4b), and nucleoredoxin 1 as the oxidoreductase protects antioxidant enzymes such as catalase from ROS-induced oxidation in plant cells^38^. In addition, *NXN* was significantly high expressed in normal tissue samples compared with colon adenocarcinoma (COAD) and rectum adenocarcinoma (READ) cases (Fig. 4d). Similarly, *Parvimonas micra* is enriched in colorectal cancer^1^, its associated host gene *PARVB* (Parvin Beta, rs35928604, *P* = 1.55 × 10^−8^, Fig. 4c) was overexpressed in colorectal cancer including COAD and rectum adenocarcinoma (READ) (Fig. 4e), which supported the previous study that reported overexpression of *PARVB* correlated significantly with lymph node metastasis and tumor invasion^39^. *Bifidobacterium dentium*, *Parvimonas micra* and *Porphyromonas asaccharolytica* are all bacteria found in the human oral cavity that are normally in low abundance in the colon. These findings are consistent with the notion that immune defense are important drivers of host-microbiome co-evolution in addition to metabolism.

## Discussion

The present study performed a comprehensive M-GWAS analysis integrating a total of 1295 host whole-genome and fecal whole metagenome sequencing to investigate the associations between genetic variants and gut microbiome in Chinese adults. Using common variants-, rare variants-, and CNVs-based association analysis without loosening the *p* value cutoff, we identified 37 loci, 47 genes and 18 CNVs significantly associated with gut bacterial taxa, and they additively explained no less than 20% of the microbiome composition. We observed no study-wise significant associations (*P* < 1 × 10^−10^ for over 500 taxa) in this study. However, consistent with previous M-GWAS from Germany, the Netherlands and Israel^7,9,10^, abundant signals were only detected with genome-wide significance. Furthermore, a meta-analysis of tens of thousands of individuals of mostly European origins only identified study-wide significance in *LCT* locus^40^. We refrained from reporting more suggestive associations except for the enterotype results. More recently, M-GWAS using microarray and amplicon data of 1475 individuals from Guangzhou city of China^41^ found 11 SNPs significantly associated with taxa in the discovery stage before adjustment (*P* < 5 × 10^−8^). We could replicate 5 of the 11 SNPs at phylum level (*P* < 0.05). These identified M-GWAS signals need to be replicated in more independent Chinese samples

Notably, although insufficient power to detect variants (rare variants and CNVs etc.) in low-depth sequencing data, we still replicated our key findings for “enterotypes”, T2D-KOs, common variants' associations, gender-differential associations, and MWAS markers (Figs. 1–4, Supplementary Tables S9 and S22). The majority of associations lie in metabolic, neurological and immunological functions, which is particular interesting considering the rapid changes in lifestyle and environmental factors in China and the rising disease incidences. For example, a good portion of our Chinese cohort still harbor *Prevotella* instead of *Bacteroides*, compared with western country^17^. To investigate the effect of host genome on enterotype, we identified two suggestive loci explaining 11% of the *Prevotella–Bacteroides* variances. These two tentative associations are not yet genome-wide significant (*P*_*P-B*_ = 2.08 × 10^−6^ and *P*_*P-B*_ = 2.6 × 10^−6^, respectively, using *Prevotella* as cases and *Bacteroides* as controls in logistic regression model), but we feel obliged to report them after replication, given the long-lasting arguments in multiple studies^14,42,43^ over the concept of “enterotypes”. It is intriguing that heterozygous individuals show two clusters of either high or low *Prevotella* (Fig. 1). In addition, we identified heritability and specific loci for *Prevotella* species; the minor allele T of rs1453213 at *OXR1* was consistently correlated with higher abundance of family Prevotellaceae and *Prevotella* species, and higher frequency of allele T in Asian population (*f* = 0.39) than European population (*f* = 0.28) may also explain the enrichment of *Prevotella* in Asian in addition to the diet. More cohorts from developing countries in the future with a higher fraction of *Prevotella*-dominated individuals would help further confirm these results.

Due to the emphasis on diet in early studies and recently on medication, MWAS^1,31^ have received even more controversy than GWAS. Besides diet and medication, we also took into account physical activity in this 4D-SZ cohort^44,45^. Here we find that fecal biomarkers previously reported by MWAS studies on colorectal cancer and metabolic diseases have some associations with host genetics, whereas some taxa especially some spore-forming bacteria lacked host genetic associations. With 1 liter of saliva swallowed every day, genetically encoded responses to ectopic presence of oral bacteria in the gut may be a common theme in a number of diseases investigated by MWAS, as has been shown for inflammatory bowel disease^46^.

Gender stratification GWAS could be used to identify novel loci that may have been previously undetected in gender-combined GWAS and had been performed in human complex traits^13,47^, whereas none had done it for gut microbiome. Here, we performed the first gender-specific M-GWAS and identified 33 male-specific (involved in inflammation, such as SLE and leishmania infection) and 37 female-specific associations (involved in olfactory signaling and GPCR signaling) linked to gut bacteria by gender-specific analysis, suggesting the importance of discriminating gender in M-GWAS and it will help better understand the underlying molecular mechanisms between genders. In summary, our results first reveal the influence of host genome on gender-differential gut bacteria and remind researchers to consider the effect of community types and gender stratification in the meta-analysis of the heterogeneous large population.

## Materials and methods

### Cohort descriptions

632 individuals were enlisted in the discovery cohort and 663 individuals were enlisted in the replication cohort, as part of the larger effort of 4D-SZ study^44,45^. Questionnaires were collected through a cell phone application. After excluding individuals that were pregnant, taking antibiotics within one month or suffering from diseases, 620 individuals in the discovery cohort and 663 individuals in the replicate cohort were remained. All participants provided blood samples during physical examination. The MGIEasy stool collection kit containing a room temperature stabilizing reagent that preserves metagenomic samples^48^, were also given to the volunteers, who handed in fecal samples on the same morning or the day after. All samples were retrieved from the boxes in front of restrooms and then stored at −80 °C before DNA extraction. For blood sample, buffy coat was isolated and DNA was extracted using HiPure Blood DNA Mini Kit (Magen, Cat. no. D3111) according to the manufacturer’s protocol. Feces were collected by MGIEasy and stool DNA was extracted in accordance with the MetaHIT protocol^31^ as described previously. The DNA concentrations from blood and stool samples were estimated by Qubit (Invitrogen). In all, 500 ng of input DNA from blood and stool samples were used for library formation and then processed for single-end 100 bp sequencing on BGISEQ-500 platform^49^.

The study was approved by the Institutional Review Boards (IRB) at BGI-Shenzhen, and all participants provided written informed consent at enrollment.

### High-depth WGS alignment and SNP/INDEL calling in the discovery cohort

Whole-genome reads were aligned to latest reference human genome GRCh38/hg38 with BWA^50^ (version 0.7.15) with default parameters. The reads consisting of base quality <5 or containing adaptor sequencing were filtered out. The alignments were indexed in the BAM format using Samtools^51^ (version 0.1.18) and PCR duplicates were marked for downstream filtering using Picardtools (version 1.62). The Genome Analysis Toolkit’s (GATK^52^, version 3.8) BaseRecalibrator created recalibration tables to screen known SNPs and INDELs in the BAM files from dbSNP (version 150). GATKlite (v2.2.15) was used for subsequent base quality recalibration and removal of read pairs with improperly aligned segments as determined by Stampy. GATK’s HaplotypeCaller were used for variant discovery. GVCFs containing SNVs and INDELs from GATK HaplotypeCaller were combined (CombineGVCFs), genotyped (GenotypeGVCFs), variant score recalibrated (VariantRecalibrator), and filtered (ApplyRecalibration). During the GATK VariantRecalibrator process, we took our variants as inputs and used four standard SNP sets to train the model: (1) HapMap3.3 SNPs; (2) dbSNP build 150 SNPs; (3) 1000 Genomes Project SNPs from Omni 2.5 chip; and (4) 1000 G phase1 high confidence SNPs. The sensitivity threshold of 99.9% to SNPs and 99% to INDELs were applied for variant selection after optimizing for Transition to Transversion (TiTv) ratios using the GATK ApplyRecalibration command. After applying the recalibration, there are 43,342,216 raw variants left, including 38 million SNPs, 5 million INDELs.

We applied a conservative inclusion threshold for variants: (i) mean depth >8 ×; (ii) Hardy-Weinberg equilibrium (HWE) *P* > 10^−5^; and (iii) genotype calling rate >98%. We demanded samples to meet these criteria: (i) mean sequencing depth >20 ×; (ii) variant call rate >98%; (iii) no population stratification by performing principal components analysis (PCA) analysis implemented in PLINK^53^ (version 1.07) and (iv) excluding related individuals by calculating pairwise identity by descent (IBD, Pi-hat threshold 0.1875) in PLINK. Only 2 samples were removed in quality control filtering and 618 individuals entered into subsequent analysis.

### CNV calling

The CNV call set were produced using the SpeedSeq^54^ pipeline, followed by the svtools package (v0.2.0; https://github.com/hall-lab/svtools). In brief, speedseq sv, which comprises LUMPY for SV calling based on discordant pairs and split-reads; svtyper for SV genotyping; and cnvnator for read-depth based CNV detection; was run on each sample individually. The individual-level calls were sorted and merged using svtools lmerge, and then each sample was re-genotyped and copy number annotated at all variant positions using svtools genotype and copy number, and pasted into a single cohort-level VCF. For filtering, inversion calls and adjacencies (i.e., BNDs) were excluded. The CNV was defined as known in the DGV^29^ (http://projects.tcag.ca/variation) if it had 70% region overlapped with one CNV in DGV.

### Low-depth WGS alignment and SNP/INDEL calling in the replicate cohort

We used BWA to align the whole-genome reads to GRCh38/hg38 and used GATK to perform variants calling by applying the same pipelines for high-depth WGS data. After finishing the GenotypeGVCFs process, we got 29,906,793 raw variants. A more stringent process (hard filter not VQSR) in the GATK VariantRecalibrator stage compared with high-depth WGS was then used, as are recommended for low-coverage whole-genome data, to filter the uncertain genotype calls and keep only high-quality variants. Specifically, we excluded individual SNPs with low mapping quality (*Q* < 20) and SNPs with low depth (DP < 3). Then we kept variants with <30% missing information. Since alleles at lower frequencies are less informative for association analysis, we excluded from downstream analysis SNPs that are at frequency of less than 0.5% in our sample, leaving 779,521 highly reliable variants. All these high-quality variants were then imputed using BEAGLE 5^55^ with 618 high-depth WGS data set as reference panel. We retained only variants with imputation information >0.7 and got 5,318,809 imputed variants. Finally, we further filtered this set to keep variants with Hardy–Weinberg equilibrium *P* > 10^−5^ and genotype calling rate >90%, yielding 5,249,443 variants for subsequent analysis.

To evaluate the data quality, we sequenced 27 samples with both high-depth and low-depth WGS data and then compared the 5,318,809 variants between them for each individual. The average genotype concordance was 98.66% (Supplementary Table S24).

### Metagenomic profiling

There were mainly two steps for metagenomic profiling: (1) computation of relative gene abundance. The high-quality metagenomic sequencing reads were first aligned to human genome hg38 using SOAP2^56^ (version 2.22). Human (host) reads were removed if the criterion of identity ≥90% in alignment. Then, high-quality reads were aligned against integrated gene catalog (IGC)^57^ by SOAP2 using the criterion of identity ≥95%. To eliminate the influence of sequencing amount in comparison analyses, we downsized the unique IGC mapped reads to 20 million for each sample. After reads aligning to gene, the gene abundance profiling was determined as previously described^31^. (2) Construction of gut taxa, KO and GMM profiles. For the species profile, we used phylogenetic assignment of each gene from the original gene catalog and summed the relative abundance of genes from the same species to yield the abundance of that species. Relative abundance of each species in a sample constituted the species profile of that sample. The relative abundance profiles of phylum, order, family, class, genus and KEGG^58^ orthologous groups (KOs) were determined from the gene abundances in the same method. In addition, GMMs reflect bacterial and archaeal metabolism specific to the human gut, with a focus on anaerobic fermentation processes^27^. The current set of 103 GMMs was built through an extensive review of the literature and metabolic databases, inclusive of MetaCyc^59^ and KEGG, followed by expert curation and delineation of modules and alternative pathways. We identified 98 common GMMs present in 50% or more of the samples. The code about metagenomic profile construction was also shared in github: https://github.com/Scelta/cOMG.

### Covariates used in this study

As part of the 4D-SZ cohort, all participants in this study had records of multi-omics data, including anthropometric measurement, stool form, defecation frequency, diet, lifestyle, blood parameters, hormone, etc.^44^. We tested for associations between these environmental factors and microbiome β-diversity at the genus level. The effect size (R-square) and significance of the mentioned variables were estimated using both “envfit” function and “capscale” function in vegan (R 3.2.5, vegan package 2.4-4). The two methods produced the consistent results that gender, BMI, defecation frequency and the lifestyle of stay up late were the strongest factors to explain gut microbiome composition (Supplementary Table S2). In addition, given the effects of diet and lifestyles on specific taxa, we finally included age, gender, BMI, defecation frequency, stool form, 12 diet and lifestyle factors, as well as the top four principal components (PCs) as covariates for subsequent M-GWAS analysis.

### Enterotype analysis

The enterotypes analysis was performed using genus-level gene abundance data according to the DMM-based clustering approach^60,61^ and two enterotypes were identified among the 618 healthy Chinese individuals in discovery cohort, including *Bacteroides* (enterotype 1, *n* = 440) and *Prevotella* (enterotype 2, *n* = 178). Using the same method, this replicate cohort comprised of 473 *Bacteroides*-dominated and 190 *Prevotella*-dominated individuals. We used logistic model implemented in PLINK to run a GWAS for genetic variation and the enterotype phenotype (i.e., *Bacteroides* and *Prevotella*; dichotomous trait). We estimated the proportion of enterotypes’ variance explained by top two loci using the restricted maximum likelihood method implemented in GCTA.

### Association analysis for microbiome β-diversity

The microbiome β-diversity (between-sample diversity) based on genus-level abundance data were generated using the “vegdist” function (Bray–Curtis dissimilarities). Then, we performed PCoA based on the calculated beta-diversity dissimilarities using the “capscale” function in “vegan”. The associations between genetic variants and microbiome β-diversity was performed using microbiomeGWAS^62^ tool.

### Genome-wide association analysis for gut bacteria

We tested the associations between host genetics and gut bacteria using linear or logistic model based on the abundance of gut bacteria. The abundance of bacteria appeared in over 95% of individuals was transformed by the natural logarithm and the outlier individual who was located away from its mean by more than five standard deviations was removed, so the abundance of bacteria could be treated as quantitative trait. Otherwise, we dichotomized bacteria into presence/absence patterns to prevent zero inflation, then the abundance of bacteria could be treated as dichotomous trait. More specifically, a total of 718 gut taxa present in over 10% individuals were analyzed in this study. The 331 taxa that appeared in over 95% of individuals were used as quantitative traits, and the other 387 taxa that appeared in <95% of individuals but over 10% individuals were used as binary traits (Supplementary Table S8).

Next, for the common variants with MAF > 5%, we performed a standard single variant (SNP/INDEL)-based GWAS analysis via PLINK using a linear model for quantitative trait or a logistic model for dichotomous trait, a threshold of *P* < 5 × 10^−8^ was used for genome-wide significance. We used the same methods for CNVs-based association analysis and set a significance threshold at *P* < 6.25 × 10^−6^ accounting for 8006 common CNVs (MAF > 1%). For rare variants-based association analysis, we applied the Sequence Kernel Association Test^63^ (SKAT) to the rare variants (MAF ≤ 5%) for each gene. Gene regions were annotated using the RefSeq^64^ database with a total of 27,874 genes. We only included the genes, which had five or more rare variants (as recommended by the SKAT authors) for testing; 22,015 genes satisfied this requirement. Associations were considered significant with *P* < 2.14 × 10^−6^ (equal to 0.05/22,015). When testing all the association analysis, we adjusted for gender, BMI, defecation frequency, stool form, self-reported diet, lifestyle factors and the first four PCs.

Gene-based analysis identified 40 genes for microbial taxa. To quantify the fraction of microbiome variance that could be inferred from gene-based analysis (actually rare variants), we first selected 200 top-ranking rare variants (not in linkage equilibrium) according to their association with taxa, then performed a greedy stepwise algorithm, in which at each iteration we added the most significant variant to the inferred variant sets added in previous iterations. Before adding each variant, we performed 1000 permutation tests and verified that its contribution was greater than in at least 50% of these permutations using “capscale” function. If not, we stopped the algorithm. In each permutation, we assigned the top 200 rare variants of each individual to a random individual, and then reran the entire analysis. Finally, 60 loci were used to infer the variance of rare variants explained for microbial composition. For 37 loci from common variants-based association analysis, 60 loci from above rare variants analysis and 18 loci from CNVs-based association analysis, the effect of each significant loci on genus-level composition was determined using bray-distance based redundancy analysis (“capscale” in the “vegan” package in R). After calculating the contributions of each significant loci on genus-level composition, we estimated the additive effects of these significant loci on genus-level composition using the “ordiR2step” function in the “vegan” package in R. The ordiR2step function performs forward model choice solely on adjusted *R*^2^. The adjusted *R*^2^ of the model including 37 significant common variants was calculated as the variance explained by common variants for microbial composition. The adjusted *R*^2^ of the model including 60 significant rare variants was calculated as the variance explained by rare variants for microbial composition. The adjusted *R*^2^ of the model including 18 significant CNVs was calculated as the variance explained by CNVs for microbial composition. The adjusted *R*^2^ of the model including all 115 significant variants was calculated as the variance explained by host genetics for microbial composition.

### Functional annotation of significant loci

Genome-wide significant loci identified in M-GWAS were mapped to genes using SNP2GENE in FUMA^26^ (http://fuma.ctglab.nl/). We first converted the loci positions from hg38 to hg19, then used the positional mapping method and maps variants to genes based on physical distance within a 20 kb window. Mapped genes were further investigated using the GENE2FUNC procedure, which provides hypergeometric tests of enrichment of the list of mapped genes in 53 genotype-tissue expression (GTEx) tissue-specific gene expression sets, 7246 MSigDB gene sets, and 2195 GWAS catalog^20^ gene sets. Specifically, the background genes in the GENE2FUNC is there for the *N*, which is supposed to be all the genes we considered to select a set of interested genes n. And we have a tested gene set with m genes. The number of overlapped genes between *n* and *m* is *x*. Therefore, the null hypothesis is finding *x* genes given *N*, *n*, and *m* is not more than expected. For example, the GWAS catalog gene sets were defined by extracting genes for each trait from the GWAS catalog. Using the GENE2FUNC procedure, we examined whether the mapped genes enriched in some specific diseases or traits in GWAS catalog as well as whether enriched in specific GO, KEGG et al. The significant results were selected if Bonferroni-corrected *P* < 0.05.

### PPI network analysis

The PPI network was constructed with the Search Tool for Retrieval of Interacting Genes/Proteins (STRING^65^, https://string-db.org/cgi/input.pl/). Given a list of the proteins as input, STRING can search for their neighbor interactors, the proteins that have direct interactions with the inputted proteins; then STRING can generate the PPI network consisting of all these proteins and all the interactions between them. We first constructed the PPI network with the 47 significant genes as input, the network displayed on the webpage was gathered into two main clusters and then exported as a high-resolution bitmap. Meanwhile, we got the KEGG pathway enrichment results, which were used to characterize the biological importance of the clusters.

### Gender-specific GWAS analysis for microbiome

We compared the difference of diversity and microbiota composition between genders. Diversity was calculated for Shannon index based on genus-level relative abundance of microbial taxa. Pairwise comparisons were performed using non-parametric test (Wilcoxon test). The multivariate association with linear models (MaAsLin)^66^ package was used to identify the differentially abundant taxa between genders. Only taxa with *q* values < 0.05 are identified as significantly enriched in males or females.

We performed gender-specific GWAS analysis in males and females separately, by using the same methods as described in the microbiome-genome-wide association analysis. Male-specific variants were identified as (i) significantly associated with taxa in males (*P*_*male*_ < 5 × 10^−8^) and not significant in females (*P*_*female*_ > 0.05), and (ii) had nominal significant gender difference (testing *P* value for difference in gender-specific effect size estimated by beta value, *P*_*difference*_ < 0.01). Female-specific variants were identified as (i) significantly associated with taxa in females (*P*_*female*_ < 5 × 10^−8^) and not significant in males (*P*_*male*_ > 0.05), and (ii) had nominal significant gender difference (*P*_*difference*_ < 0.01, as explained below).

For each variant (SNP/INDEL/CNV) and for the phenotype (relative abundance of taxa), we computed *P* values (*P*_*difference*_) testing for difference between the male-specific and female-specific beta-estimates b_male_ and b_female_ using the T-statistic (b_male _− b_female_)/sqrt (se_male_² + se_female_² − 2*corr (b_male_, b_female_)* se_male_ * se_female_) with se_male_ and se_female_ being the standard errors of b_male_ or b_female_. The correlation between the gender-specific beta-estimates was computed as the Spearman rank correlation coefficient across all variants for each phenotype.

### Gene expression and differential analysis

We used GEPIA^67^ (Gene Expression Profiling Interactive Analysis), a web-based tool to deliver fast and customizable functionalities based on the Cancer Genome Atlas and genotype-tissue expression (GTEx) data. We performed the differential expression analysis for genes *NXN* and *PARVB* across COAD and READ types compared with paired normal samples, respectively. We choose log2(TPM + 1) transformed expression data for plotting. We used ANOVA method for differential analysis. Genes with higher |log_2_FC | > 0.2 and *p* values < 0.05 are considered differentially expressed genes.

## Supplementary information

Supplementary Figures

Supplementary Tables

## Data Availability

The data in this study have been deposited to the CNGB Nucleotide Sequence Archive (CNSA: https://db.cngb.org/cnsa; accession number CNP0000289).

## Supplementary information

**Supplementary Information** accompanies the paper at (10.1038/s41421-020-00239-w).
