# Supplementary material for: A genome-wide association study for gut metagenome in Chinese adults illuminates complex diseases
Source: Cell Discov. 2021 Feb 9;7:9. doi: 10.1038/s41421-020-00239-w (PMC7873036; doi:10.1038/s41421-020-00239-w)
Supplement: Supplementary file 1 — Supplementary Figures [file 41421_2020_239_MOESM1_ESM.pdf]

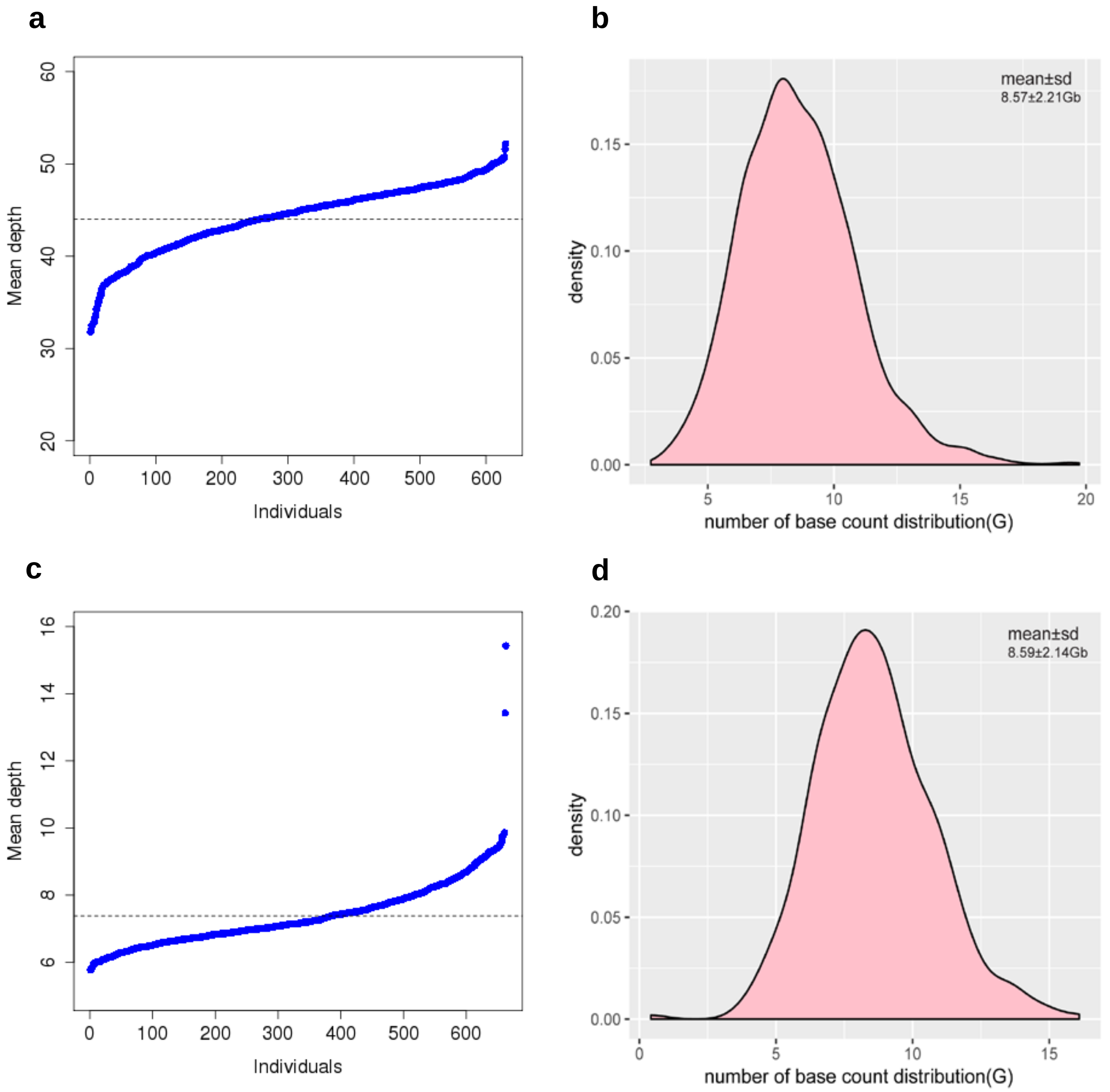

Supplementary Fig. S1. Whole genome and metagenome sequencing data production. **a,** Depth distribution of 632 high-depth WGS samples in discovery cohort. The mean depth is 44×. **b,** Metagenome sequencing at an average of 8.57±2.21 Gb per sample in discovery cohort. **c,** Depth distribution of 663 low-depth WGS samples in replication cohort. The mean depth is 7×. **d,** Metagenome sequencing at an average of 8.59±2.14 Gb per sample in replication cohort.

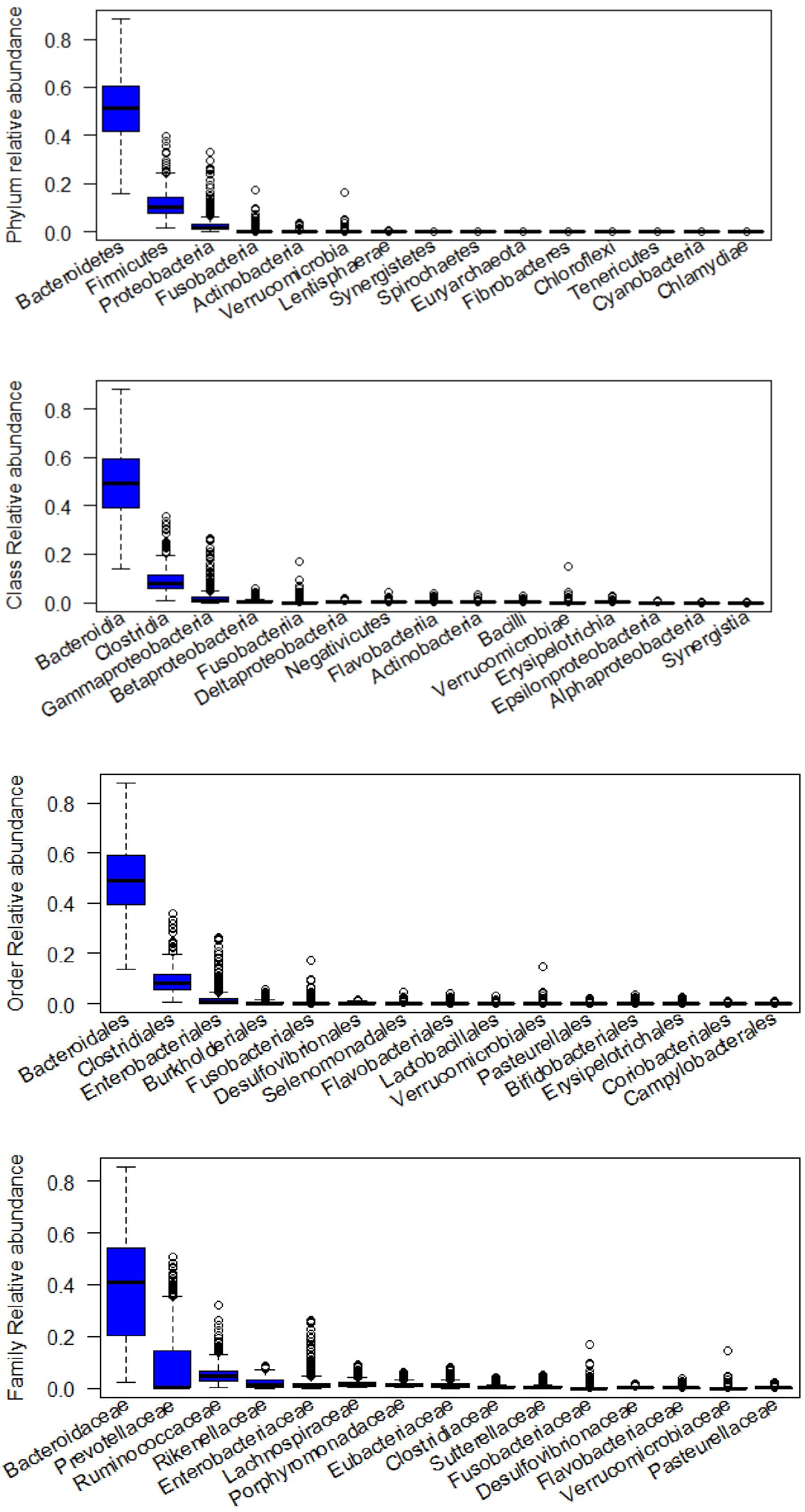
Phylum relative abundance
0.0
0.2
0.4
0.6
0.8
Bacteroidetes
Firmicutes
Proteobacteria
Fusobacteria
Actinobacteria
Verrucomicrobia
Lentisphaerae
Synergistetes
Spirochaetes
Euryarchaeota
Fibrobacteres
Chloroflexi
Tenericutes
Cyanobacteria
Chlamydiae
Class Relative abundance
0.0
0.2
0.4
0.6
0.8
Bacteroidia
Clostridia
Gammaproteobacteria
Betaproteobacteria
Fusobacteria
Deltaproteobacteria
Negativicutes
Flavobacteria
Actinobacteria
Bacilli
Verrucomicrobiae
Erysipelotrichia
Epsilonproteobacteria
Alphaproteobacteria
Synergistia
Order Relative abundance
0.0
0.2
0.4
0.6
0.8
Bacteroidales
Clostridiales
Enterobacteriales
Burkholderiales
Fusobacteriales
Desulfovibrionales
Selenomonadales
Flavobacteriales
Lactobacillales
Verrucomicrobiales
Pasteurellales
Bifidobacteriales
Erysipelotrichales
Coriobacteriales
Campylobacterales
Family Relative abundance
0.0
0.2
0.4
0.6
0.8
Bacteroidaceae
Prevotellaceae
Ruminococcaceae
Rikenellaceae
Enterobacteriaceae
Lachnospiraceae
Porphyromonadaceae
Eubacteriaceae
Clostridiaceae
Sutterellaceae
Fusobacteriaceae
Desulfovibrionaceae
Flavobacteriaceae
Verrucomicrobiaceae
Pasteurellaceae

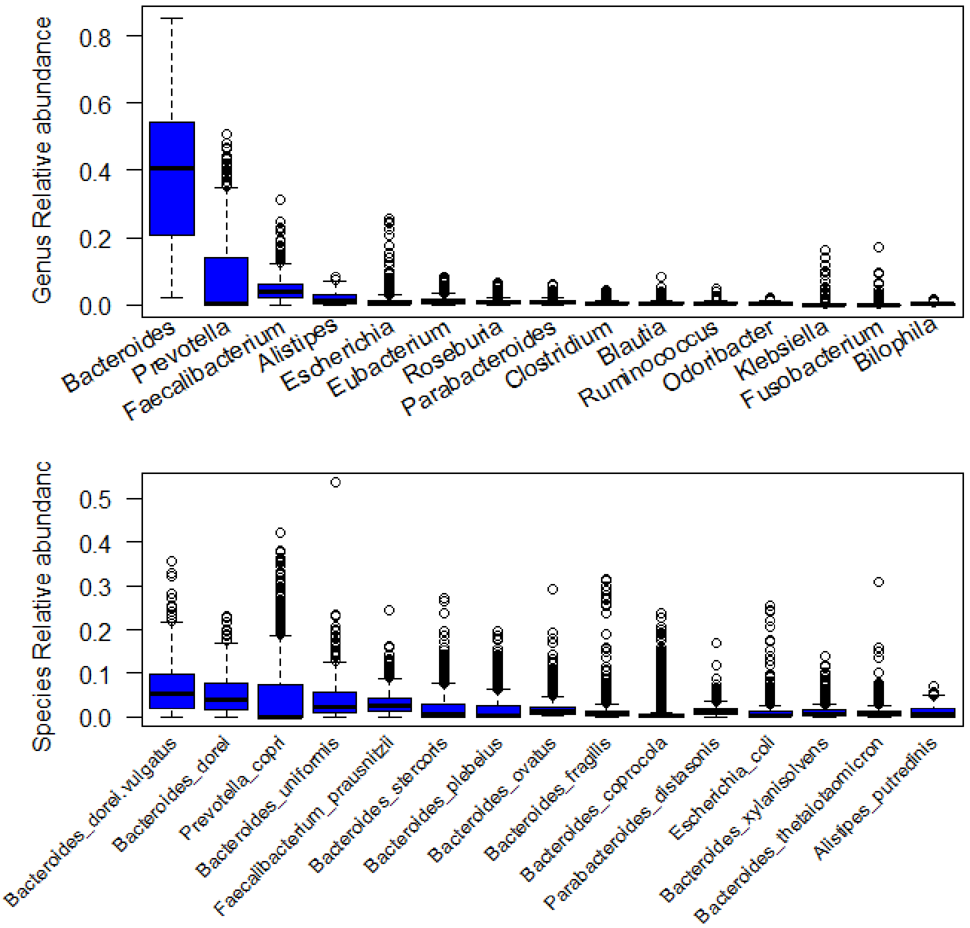

Supplementary Fig. S2. Box and whisker plot of the top 15 taxa with high relative abundance from phylum to species level in the Chinese cohort. Boxes represent the interquartile range (IQR) between first and third quartiles and the line inside represents the median. Whiskers denote the lowest and highest values within 1.5 × IQR from the first and third quartiles, respectively. Circles represent outliers beyond the whiskers**.**

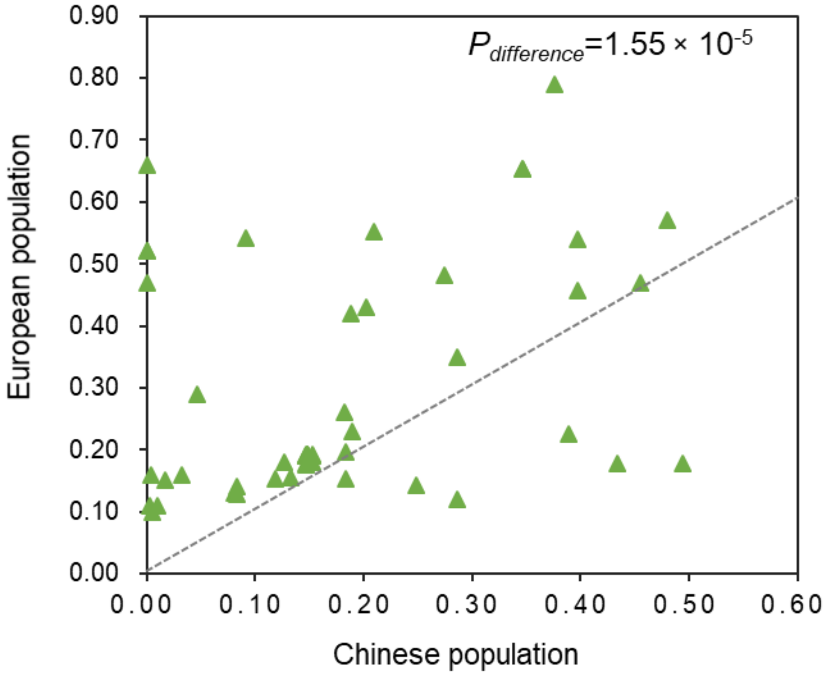

Supplementary Fig. S3. The alleles frequencies difference of reported 64 loci associated with beta-diversity between two populations. The European population represents the EUR population in 1000genome phase3, and the alleles frequencies were acquired by searching in Haploreg website (https://pubs.broadinstitute.org/mammals/haploreg/haploreg.php).

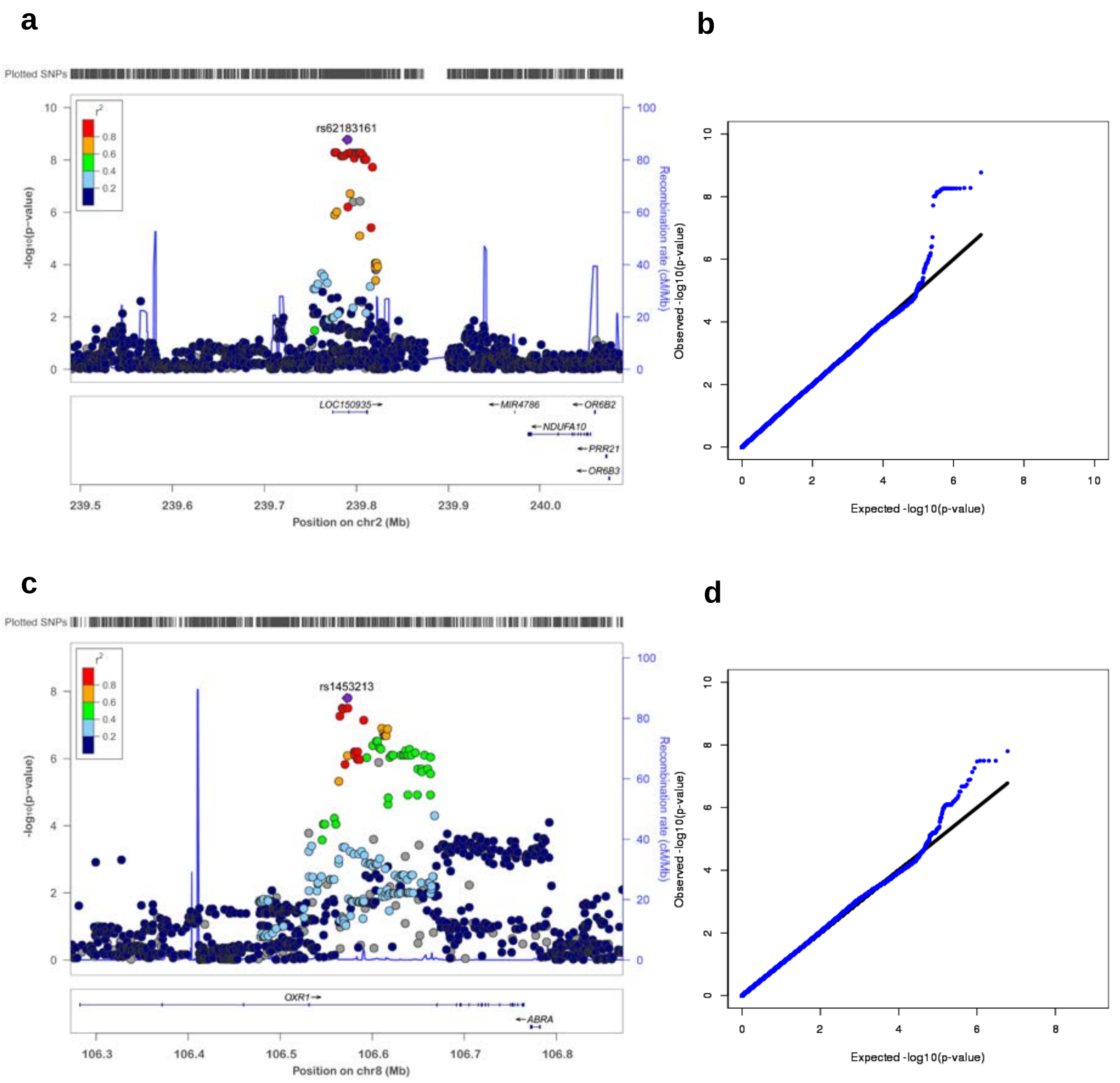

Supplementary Fig. S4. Regional and Quantile-quantile (QQ) plots showing the associations between two top loci and taxa. a, Regional plot of *LOC150935* associated with phylum Actinobacteria, the index SNP rs62183161 (P=1.68 × 10^{-9}) was showed in purple rhombus. **b,** QQ plot with observed –log10 (p values) and the expected –log10 (p values) for phylum Actinobacteria. The genomic inflation factor is 1.019 (λ=1.019). **c,** Regional plot of *OXR1* associated with family Prevotellaceae, the index SNP rs1453123 (P= 1.58 × 10^{-8}) was showed in purple rhombus. **d,** QQ plot with observed –log10 (p values) and the expected –log10 (p values) for family Prevotellaceae. The genomic inflation factor is 1.018 (λ=1.018).

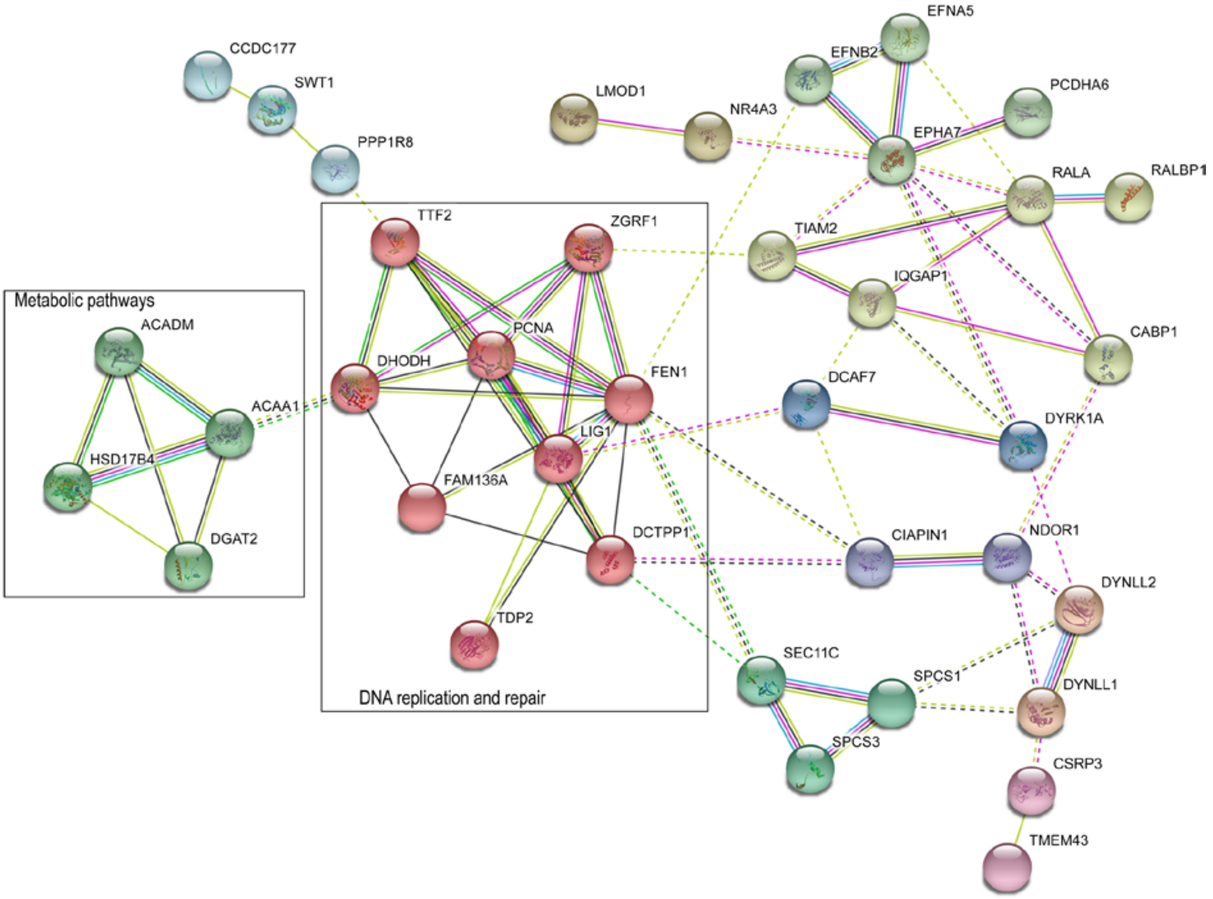

Supplementary Fig. S5. Protein-protein interaction analyses. The network contains 34 proteins and their 5 connected protein. Stronger associations are represented by thicker lines. Enrichment p-value=0.037. Two main pathways were marked with black rectangles, including metabolic pathways and DNA replication and repair pathways.

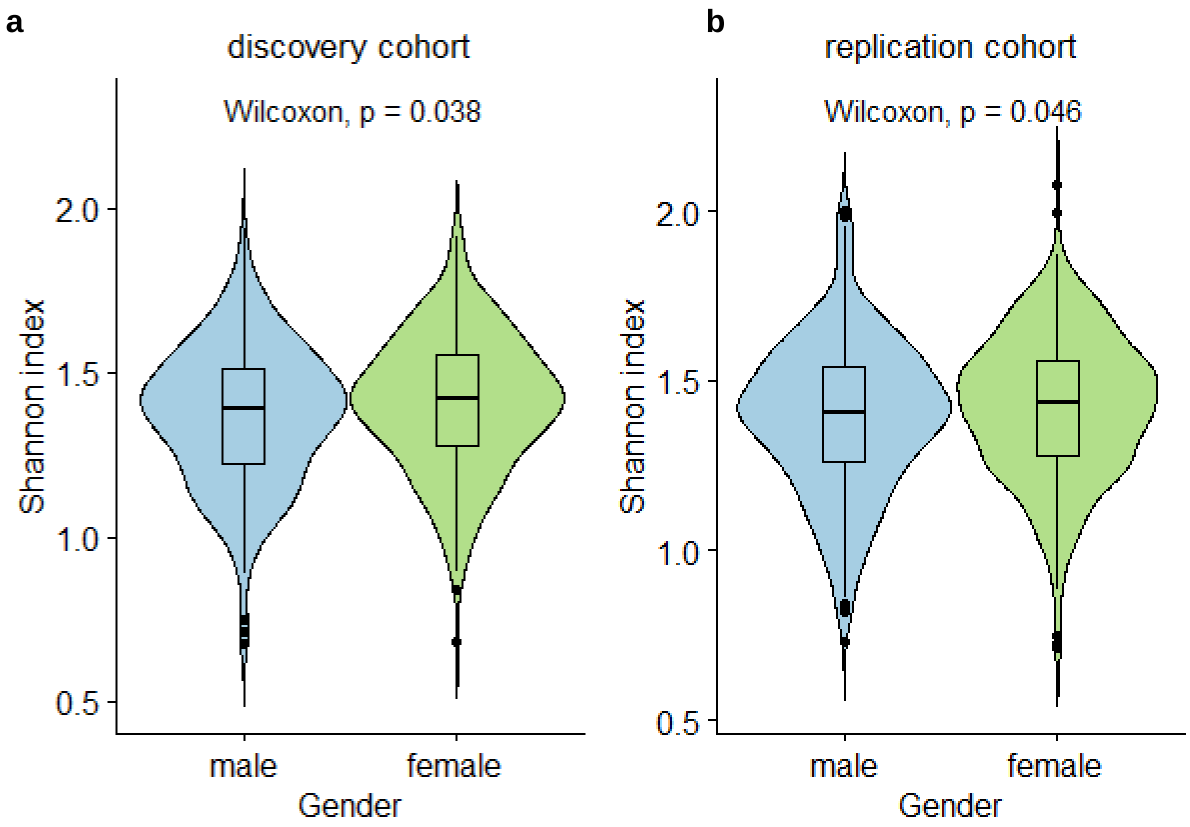

Supplementary Fig. S6. Violin plots of sex differences on alpha diversity. Alpha diversity was calculated for Shannon index based on genus-level relative abundance of taxa. Pairwise comparisons were performed using non-parametric test (Wilcoxon test). Both discovery **(a)** and replication **(b)** cohorts showed sex differences (Wilcoxon *P*=0.038 and *P*=0.046, respectively).
